# Dipeptidyl Peptidase-4 Regulation of SDF-1/CXCR4 Axis: Implications for Cardiovascular Disease

**DOI:** 10.3389/fimmu.2015.00477

**Published:** 2015-09-25

**Authors:** Jixin Zhong, Sanjay Rajagopalan

**Affiliations:** ^1^Division of Cardiovascular Medicine, University of Maryland, Baltimore, Baltimore, MD, USA

**Keywords:** SDF-1, CXCR4, dipeptidyl peptidase-4, cardiovascular, chemokine

## Abstract

Dipeptidyl peptidase-4 (DPP4) is a ubiquitously expressed protease that regulates diverse number of physiological functions. As a dipeptidase, it exerts its catalytic effects on proteins/peptides with proline, alanine, or serine in the penultimate (P1) amino acid residue from the amino terminus. The evidence to date supports an important effect of DPP4 in catalytic cleavage of incretin peptides and this perhaps represents the main mechanism by which DPP4 inhibition improves glycemic control. DPP4 also plays an important role in the degradation of multiple chemokines of which stromal cell-derived factor-1 (SDF-1, also known as CXCL12) is perhaps an increasingly recognized target, given its importance in processes, such as hematopoiesis, angiogenesis, and stem cell homing. In the current review, we will summarize the importance of DPP4-mediated enzymatic processing of cytokines/chemokines with an emphasis on SDF-1 and resultant implications for cardiovascular physiology and disease.

## Introduction

Dipeptidyl peptidase-4 (DPP4) is a type-II integral transmembrane glycoprotein that has recently gained attention owing to its role in the catalytic degradation of incretins and as a receptor for entry for the Middle Eastern respiratory syndrome (MERS) virus. DPP4 is best known for its catalytic function, whereby it proteolytically cleaves a number of peptide and protein substrates. It exhibits a strong preference for peptides with proline, serine, or alanine as the penultimate amino acid from the amino terminus. The N-terminus of glucagon insulotropic peptide (GIP) and glucagon-like peptide-1 (GLP-1) consist of Tyr–Ala and His–Ala, respectively, rendering them excellent substrates for DPP4. In addition to its role in modulation of incretin peptides, such as GLP-1 and GIP, DPP4 regulates immune responses via cleavage of many cytokines and chemokines, including stromal cell-derived factor-1 (SDF-1, also known as CXCL12), involved in immune function and physiological functions, such as angiogenesis. In this review, we will focus on the regulatory aspects of DPP4 on chemokines, such as SDF-1, and its potential implications in the pathogenesis and management of cardiovascular disease.

## An Overview of DPP4 Biology

Dipeptidyl peptidase-4 also called cluster of differentiation-26 (CD26) or adenosine deaminase-binding protein (ADA-binding protein), is a member of S9b peptidases. The S9b family consists of structurally homologous proteolytic enzymes, including DPP2 (also called quiescent cell proline dipeptidase, QPP), DPP4, DPP8, DPP9, and fibroblast activation protein (FAP). DPP4 was first identified as a new dipeptide naphthylamidase in 1966 (1) and subsequently found to be identical to T cell activation antigen CD26, ADA-binding protein, mouse thymocyte-activating molecule, and rat liver membrane glycoprotein gp110 (2). DPP4 consists of a short N-terminal intracellular domain (6 residues), a 22-residue-long transmembrane α-helix domain (23 amino acids), and a large C-terminal extracellular domain. The C-terminal extracellular domain is responsible for its catalytic activity and binding to a number of ligands, such as ADA and matrix proteins (3). The catalytic activity of DPP4 depends on its dimerization and glycosylation of specific residues (2). DPP4 can also assemble into tetramers on the cell surface, which may involve the linkage of dimers located on the surface of two different cells, enabling it to function as a cell–cell communication molecule. In addition to its membrane-bound form, DPP4 also circulates as a soluble form in the plasma, which lacks the cytoplasmic and transmembrane domain with preserved catalytic activity. Soluble DPP4 (sDPP4) is a homodimer with a molecular weight range of 210–290 kDa (4), but can form higher molecular weight assemblies migrating as 900-kDa complexes (5). Whether sDPP4 is cleaved from the membrane or is secreted is unclear. For instance, studies investigating viral liver infection suggested that sDPP4 is shed from membrane-bound DPP4 (6). sDPP4 has, however, also been detected in the lumen of secretory granules in pancreatic α cells and in the exocytic secretory lysosomes of natural killer cells (7, 8). sDPP4 is commonly elevated in many disorders, such as solid tumors, reactive airways disease, hepatitis C, type 2 diabetes, and obesity (6, 9, 10).

### Tissue distribution and cell specific expression and phenotype of *Dpp4*^−/−^ mice

Dipeptidyl peptidase-4 is widely distributed throughout the body (Table 1), with particularly high expression on the apical surface of endothelial and differentiated epithelial cells. Bone marrow cells, brush border of the small intestine, proximal tubular cells, and glomerular cells in the kidney express high levels of DPP4 as well (11). DPP4 is present on endothelial cells and fibroblasts throughout the body. Among hematopoietic cells, DPP4 is expressed at the highest level on T cells with lower levels in monocytes and dendritic cells (12). DPP4 expression increases as monocytes differentiate into antigen-presenting cells as well as during T cell activation (12, 13). DPP4 is expressed at high levels in kidney, spleen, lung, pancreas, and prostate (14). Mice lacking the gene encoding DPP4 are refractory to the development of obesity and hyperinsulinemia and demonstrate improved post-prandial glucose control (15, 16). Mice deleted for *Dpp4* are fertile and appear healthy. Only slight decrease of body weight in *Dpp4*^−/−^ mice was observed compared to wild types. They have normal fasting blood glucose level, but shows reduced glycemic excursion after an oral glucose challenge (16). Increased intact insulinotropic form of GLP-1 and circulating insulin were seen in *Dpp4*^−/−^ mice after oral glucose stimulation (16). Pair feeding and indirect calorimetry studies indicate that reduced food intake and increased energy expenditure accounted for the resistance to high fat diet-induced obesity in the *Dpp4*^−/−^ mice. Ablation/deletion of DPP4 is associated with improved metabolic control with improved insulin sensitivity, reduced pancreatic islet hypertrophy, and protection against streptozotocin-induced loss of β cell mass and hyperglycemia (15). Pharmacological inhibition of DPP4 enzymatic activity improves glucose tolerance in wild-type but not in *Dpp4*^−/−^ mice. Interestingly, DPP4 inhibitor’s also improve glucose tolerance in *Glp1r*^−/−^ mice, indicating that DPP4 contributes to blood glucose regulation by controlling the activity of GLP-1 as well as additional substrates (16).

**Table 1:** **Major distribution and potential function of DPP4**.

Distribution	Potential function
Adipocyte	Serves as an adipokine mediating obesity-induced metabolic syndrome (9)
Adipose tissue macrophage and dendritic cells	Enhances T cell inflammation and obesity-induced insulin resistance (12)
T cells	Promotes T cell activation by providing co-stimulatory signaling (17, 18)
Endothelial cells	Regulates endothelial function and vascular tone (19, 20)
Epithelial cells	Expressed in the epithelial cells in the kidney, lung, and GI tract. Mediates MERS-CoV infection in the lung (21), kidney fibrosis (22), diabetic nephropathy (23), intestinal growth (24)
Hepatocytes	Involved in lipogenesis (25) and liver damage (26)

## DPP4 in Cytokine Processing

Dipeptidyl peptidase-4 has been shown to be able to cleave a number of chemokines and cytokines, including SDF-1, granulocyte-macrophage colony-stimulating factor (GM-CSF), granulocyte colony-stimulating factor (G-CSF), interleukin-3 (IL-3), erythropoietin (Epo), regulated on activation normal T-cell expressed and presumably secreted (RANTES, also known as CCL5), macrophage-derived chemokine (MDC, also known as CCL22), eotaxin (also known as CCL11), monokine induced by IFN-γ (MIG, also known as CXCL9), IFN-γ-induced protein-10 (IP-10, also known as CXCL10), and interferon-inducible T-cell α chemoattractant (ITAC, also known as CXCL11) (Table 2). The regulation of cytokine levels through catalytic cleavage may influence their levels in tissue domains. The differential contribution of DPP4 in the regulation of each of these cytokines is obviously dependent on the levels of expression of DPP4, which may vary depending on the tissues, the cells predominantly expressing the cytokine of interest and the disease context. Additionally, circulating or cell free DPP4 may also contribute to catalytic activity. DPP4-mediated truncation of RANTES abolishes the chemotactic activity to monocytes but not to T cells (27). Eotaxin (CCL11) is an eosinophil chemotactic protein and has been shown to be involved in allergic responses. DPP4 truncation of eotaxin inactivates its chemotactic activity for eosinophils. DPP4-truncated eotaxin (3–74) shows impaired binding and signaling through CCR3. In addition, truncated eotaxin suppresses calcium signaling and chemotaxis of intact eotaxin (28). DPP4 inhibition by either genetic deletion or pharmacological inhibition, enhances eotaxin-induced mobilization of eosinophils into the blood and recruitment into the injury/injection site (29). MDC (CCL22) is a chemoattractant for monocytes, dendritic cells, NK cells, and chronically activated T lymphocytes (30, 31). DPP4-truncated MDC displays preserved chemotactic activity toward monocytes, but less potency toward lymphocytes and dendritic cells (32). CXCR3 interacts with MIG (CXCL9), IP10 (CXCL10), and ITAC (CXCL11), all of which are targets of DPP4. Cleavage of those chemokines by DPP4 attenuates their chemotactic activity, with the cleaved products serving as endogenous antagonists for CXCR3 binding (33).

**Table 2:** **Cytokine substrates of DPP4**.

Substrates	N terminal sequence	Consequence of cleavage
SDF-1 (34)	**KP**VSL…^a^	Inactivation, truncated product antagonize intact protein
RANTES (27)	**SP**YSS…^a^	Altered target cell specificity
Eotaxin (28)	**GP**ASV…^a^	Inactivation, truncated product antagonize intact protein
Erythropoietin (35)	**AP**PRL…^a^	Inactivation, truncated product antagonize intact protein
G-CSF (35)	**TP**LGP…^a^	Inactivation, truncated product antagonize intact protein
GM-CSF (35)	**AP**ARS…^a^	Inactivation, truncated product antagonize intact protein
IL-3 (35)	**AP**MTQ…^a^	Inactivation, truncated product antagonize intact protein
MDC (32)	**GP**YGA…^a^	Altered target cell specificity
IP-10 (33)	**VP**LSR…^a^	Inactivation, truncated product antagonize intact protein
MIG (33)	**TP**VVR…^a^	Inactivation, truncated product antagonize intact protein
ITAC (33)	**FP**MFK…^a^	Inactivation, truncated product antagonize intact protein

*Bold letters indicate dipeptides to be cleaved by DPP4*.

Dipeptidyl peptidase-4 is also involved in the inactivation of multiple colony-stimulating factors (CSFs) and thus regulates hematopoietic stem cells (HSCs) and hematopoietic progenitor cell (HPC) function. The proliferative action of GM-CSF, G-CSF, IL-3, and Erythropoietin (Epo) on HPCs is enhanced in DPP4 knockout mice or by pretreatment with a DPP4 inhibitor (35). Catalytic inhibition of DPP4 or DPP4 deficiency promotes the engraftment of HSCs and HPCs after bone marrow transplantation in mice (35). DPP4-truncated CSFs may suppress the activity of their respective full-length CSF via antagonism (35).

## SDF-1 and CXCR4

Stromal cell-derived factor-1 is an 8-kDa peptide that is encoded by *Cxcl12* (36). It is a chemoattractant for T lymphocytes, bone marrow stem cells [such as HSC, endothelial progenitor cell (EPC), and mesenchymal stem cells (MSCs)], endogenous cardiac stem cells (CSCs), and adipose-derived regenerative cells (37–39). There are several isoforms of SDF-1 (SDF-1α–ζ), resulting from alternative splicing of its mRNA (40). Among these isoforms, SDF-1α is the best described. SDF-1α is expressed in many tissues, including bone marrow, heart, liver, kidney, thymus, spleen, skeletal muscle, and brain (36, 40–43). In the cardiovascular system, SDF-1α is expressed in stromal cells, endothelial cells, and cardiomyocytes (44, 45). SDF-1 is typically inactivated by exopeptidases, such as DPP4, matrix metalloproteinase (MMP)-2, and -9 (34). Unlike cleavage of SDF-1 by DPP4 at position 2–3, MMPs cleave SDF-1 at position 4–5, leading to the loss of its binding activity to CXCR4 (46). The relative contribution of each of these peptidases in regulation of SDF-1 levels is unclear. CXCR4 is an alpha-chemokine receptor specific for SDF-1 and belongs to a family of G-protein-coupled receptors. CXCR4 is expressed on a range of progenitor cells (including hematopoietic, endothelial, and CSCs) and thus is important for cell migration and organ development during embryogenesis (39, 40, 47). Mice deficient for either CXCR4 or SDF-1 display abnormal B-lymphocyte, hepatic, and cardiac (ventricular septal defects) development, and die *in utero* (48–50). Loss-of-function CXCR4 mutations in humans also causes impaired neutrophil mobilization and B-cell lymphopenia (51). In addition to CXCR4, CXCR7 has also been suggested to be an important receptor for SDF-1 (52, 53). However, the relative contribution and interactions of CXCR4 and CXCR7 is not fully elucidated. The involvement of CXCR7 in cardiovascular disease, if any, is also not yet known (39). DPP4 may also play a more general role in regulating CSF activity and stem cell homing (35). It was previously believed that disruption of the interaction between CXCR4 receptor expressed by hematopoietic progenitors and SDF-1 expressed by bone marrow stromal cells is sufficient to detach anchored progenitors from their bone marrow niches, leading to their rapid mobilization to the peripheral blood. AMD3100 (also termed plerixafor) inhibits SDF-1-mediated migration *in vitro* by blocking the chemokine binding to its major receptor CXCR4 (54). AMD 3100 mobilizes immature progenitor cells from the bone marrow into the blood and has been approved for clinical mobilization in lymphoma and multiple myeloma patients undergoing autologous transplantation. When combined with G-CSF, AMD3100 synergistically augments mobilization of progenitor cells, with increased *in vitro* migration to SDF-1 gradients and facilitates repopulation of transplanted non-obese diabetic/severe combined immunodeficient mice (55). AMD 3100 has recently been shown to directly induce SDF-1 release from CXCR4^+^ human bone marrow osteoblasts and endothelial cells, with SDF-1 release from these cells into the circulation, representing a pivotal mechanism essential for steady-state egress and rapid mobilization of HPCs (56).

## DPP4 and SDF-1/CXCR4 Axis in Cardiovascular Disease

### SDF-1/CXCR4 and DPP4 inhibition in stem cell homing and engraftment

The SDF-1/CXCR4 axis has been shown to be critical in tissue repair in multiple organ systems, including the eye, heart, kidney, liver, brain, and skin. Specific to the heart, the SDF-1/CXCR4 axis has been shown to be essential for cardiogenesis (57, 58). SDF-1 is now well known as a key regulator of stem cell migration to sites of tissue injury (44, 59). SDF-1 was first identified by Askari et al. as a key regulator of stem cell migration to ischemic cardiac tissue (44). CD34^+^ stem cells express the SDF-1 receptor CXCR4 at high levels (37, 60). During myocardial infarction, SDF-1 levels are elevated 1 h after infarction and return to baseline at day 7 and further reduced to a low level thereafter (44). Overexpression of SDF-1 in ischemic cardiomyopathy by either engineered cell-based or plasmid-based approach improved cardiac function in rats via enhancing stem cell homing and promoting revascularization of the infarct area (61, 62). Therefore, the ability to express SDF-1 locally is believed to enhance the vasculogenic potential of adult cardiac progenitor cells (63). However, the enhancement of endogenous stem cell-based repair appears to be blunted due to the short half-life of SDF-1 at the time of acute myocardial infarction owing to its degradation by proteases (44). As a major enzyme mediating the degradation of SDF-1, DPP4 may represent a potential target for improving stem cell homing with stem cell-based therapy. Preservation of SDF-1 by DPP4 inhibition has been shown to promote stem cell repopulation and homing to ischemic tissues. DPP4 inhibitors diprotin A or Val–Pyr, enhance chemotaxis of HSCs and HPCs and greatly increase homing and engrafting capacity of HSCs (64, 65). Pretreatment of HSC with DPP4 inhibitor diprotin A, enhanced their repopulation ability in lethally irradiated mice (66). Enhancement of engraftment of human CD34^+^ cord blood cells with DPP4 inhibition has also been observed in xenogeneic mouse recipients (NOD/SCID or NOD/SCID/beta 2^null^) (67, 68). Pretreating either donor cells *in vitro* or recipients *in vivo* is able to enhance the engraftment of stem cells (66, 69). In a lung transplantation model, systemic DPP4 inhibition by vildagliptin increases SDF-1 levels in plasma, spleen, and lung, accompanied by a significant increase of stem cells in the lung grafts. DPP4 inhibitor-treated mice also shows less alveolar edema compared with untreated recipients (70). Liebler showed that DPP4 inhibition enhances SDF-1/CXCR4 axis and increased the retention of human bone marrow-derived cells in the injured lungs of immune deficient mice by 30% (71). In addition to SDF-1, DPP4 inhibition also enhances bone marrow engraftment by preserving G-CSF and GM-CSF. Both G-CSF and GM-CSF are substrates for DPP4, with inhibition of DPP4 promotes bone marrow engraftment not only through SDF-1 but also CSF-dependent mechanisms (35). G-CSF and GM-CSF in turn may also increase the expression of DPP4 on CD34^+^ cells, which results in their decreased responsiveness to SDF-1 (72).

### SDF-1/CXCR4 and regulation by DPP4 in angiogenesis

Angiogenesis and vasculogenesis are an immensely complex process that requires the coordinated action of a multitude of cells, transcription factors, and cytokines working in concert in a precisely choreographed manner. It is widely believed that these processes can be recapitulated in the adult through the participation of a progenitor cell population of which EPCs are perhaps the best described and widely believed to be important building blocks for the assembly of functional vasculature in adults. While the origins of EPCs are still controversial, what is clear is that these cells have the capacity to differentiate into mature endothelial cells (73). Implantation of *ex vivo*-expanded EPC has been shown to improve neovascularization of injured tissues in animal models (74–76). SDF-1 plays a pivotal role in the trafficking and homing of EPCs to ischemic tissues (77–79). SDF-1 levels increase in plasma and ischemic tissue shortly after ischemic injury, in response to hypoxia which upregulates HIF-1α (79). HIF-1α upregulates SDF-1, by binding to the promoter of SDF-1 and initiating its transcription (80). *Ex vivo* priming with SDF-1, enhances the proangiogenic potential of EPC as evidenced by improved blood flow recovery when transplanted into a nude mouse model of hind-limb ischemia (81). Disease states, such as diabetes associated with upregulation of DPP4, may represent prototypical conditions associated with defective homing and integration of EPC’s owing to rapid degradation of SDF-1 (82). Kanki et al. reported that SDF-1 could be cleaved by DPP-4 in both plasma and ischemic heart tissue (34). Shih demonstrated an improvement in EPC number and endothelial nitric oxide synthetase (eNOS) expression after DPP4 inhibition by MK-0626 (83).

### Therapeutic applications of SDF-1 and DPP4 inhibition-mediated prolongation of SDF-1 effects in cardiovascular disease

**Figure 1:**
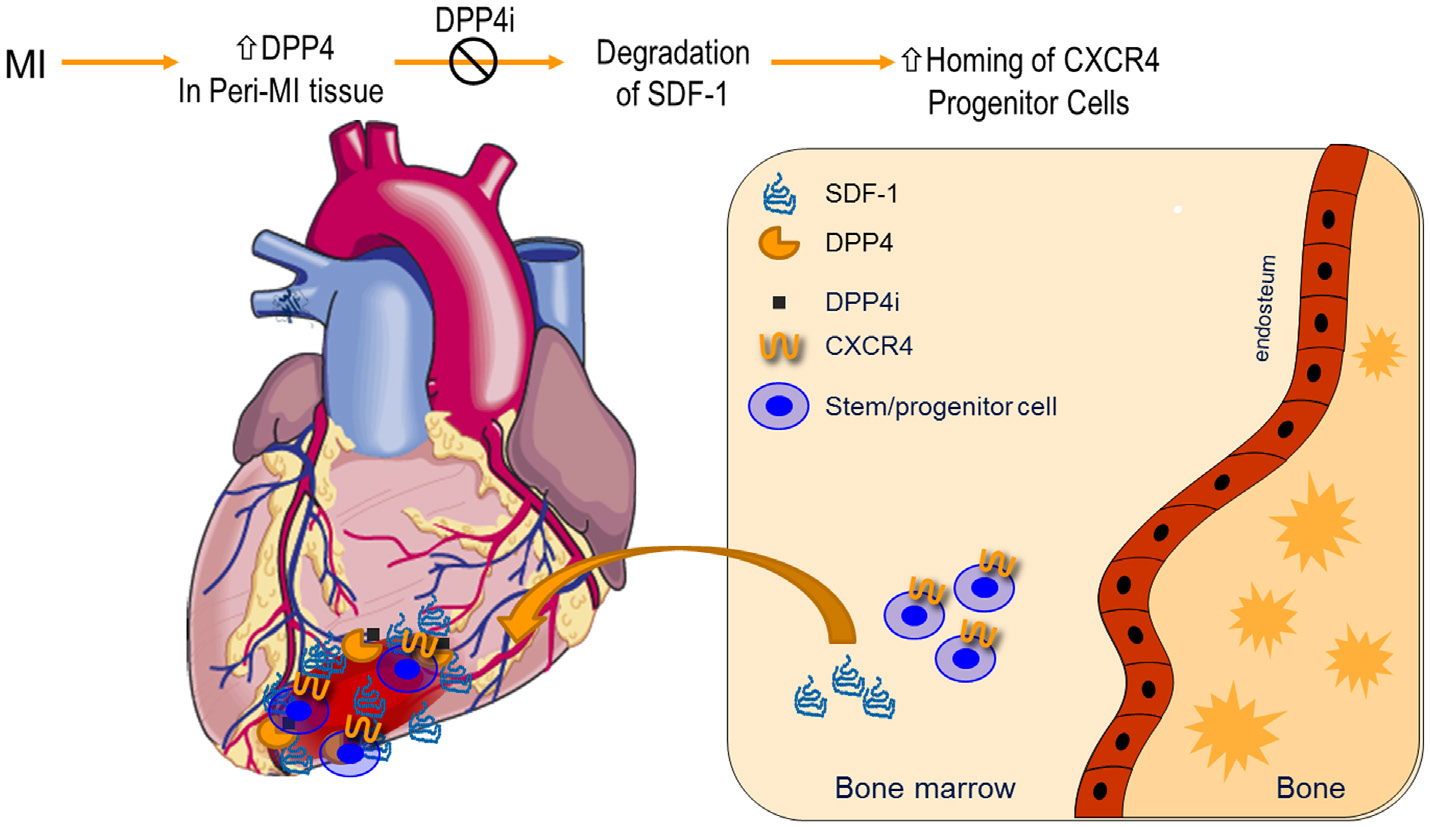
**Dipeptidyl peptidase-4 inhibition in modulation of SDF-1 and myocardial angiogenesis: expression of DPP4 increases in myocardial infarction**. Suppression of DPP4 enzymatic activities by pharmacological inhibitors preserves SDF-1, which results in an enhanced homing of CXCR4^+^ progenitor cells from bone marrow to infarcted tissues. CXCR4, chemokine (C–X–C motif) receptor 4; DPP4i, DPP4 inhibitor; MI, myocardial infarction; SDF-1, stromal-derived factor-1.

Transient engineered cell-based or plasmid-based overexpression of SDF-1 in ischemic cardiomyopathy has been shown to improve cardiac function in animal models (62). In a study that compared the effects of SDF-1 overexpressed on MSCs alone or mesenchymal stem cells engineered to overexpress SDF-1 (MSC-SDF) on cardiac function in Lewis rats after acute myocardial infarction, tail vein infusion of MSC and MSC-SDF-1, 1 day after acute myocardial infarction, led to improved cardiac function by echocardiography by 70.7 and 238.8%, respectively, compared with saline controls. The beneficial effects of MSC-SDF transplantation were suggested to be mediated through preservation rather than regeneration of cardiac myocytes within the infarct area (84). Cardiac progenitor cell and CXCR4 expression on cardiac myocytes are required for further local trophic effects of MSC (85). The mechanism of action of SDF-1 overexpression in myocardial infarction and heart failure are likely multifactorial, including both systemic and direct trophic effects. An important effect of SDF-1 is its effect on the recruitment of CSCs to the infarct and infarct border zone (59). Delivery of MSCs engineered to overexpress SDF-1 at the time of acute myocardial infarction has been shown to lead to improvement in cardiac function (61). The myocardial repair initiated by endogenous stem cell appears blunted because of the natural short-term expression of SDF-1 at the time of acute myocardial infarction. In light of these effects in regulation of SDF-1, DPP4 inhibition has been suggested to be of potential benefit in cardiovascular diseases, such as myocardial infarction and peripheral artrerial disease. In combination with G-CSF, DPP4 inhibition augments myocardial regeneration and improves cardiac function after myocardial infarction in mice (86, 87). In combination with CXCR4 overexpression, diprotin A treatment has shown to improve myocardial function and repair of infarcted myocardium (88). A bioengineered protease-resistant form of SDF-1 has shown greater potency in promoting blood flow recovery after hind-limb ischemia (89) and improving cardiac function as well as capillary density in the infarcted heart (34). Dual injection of G-CSF and sitagliptin resulted in the mobilization of progenitor cells and relieved the symptom of end-stage heart failure in a 19-month-old boy (90). Protease-resistant forms of SDF-1 display an enhanced potency in improving blood flow in experimental peripheral artery disease and myocardial infarction (34, 89). It has been shown that parathyroid hormone treatment after myocardial infarction improves survival and myocardial function with potential involvement of enhanced homing of bone marrow-derived stem cells. Huber et al. demonstrated that parathyroid hormone serves as a DPP4 inhibitor and increases cardiac SDF-1 level, which in turn enhances CXCR4^+^ bone marrow-derived stem cell homing to ischemic heart and attenuates ischemic cardiomyopathy after infarction (91). Haverslag showed SDF-1 preservation by DPP4 inhibitor increases monocyte extravasation and thus accelerating perfusion recovery without detrimental side effects on plaque stability in atherosclerosis-prone *ApoE*^−/−^ mice (92). Figure 1 depicts modulation of SDF-1 levels in the myocardium by DPP4 inhibition and enhancement of myocardial angiogenesis by DPP4 levels. In a porcine model of HF, delivery of a plasmid SDF-1 with an endomyocardial injection catheter demonstrated safety at doses up to 100 mg while improving cardiac function and vasculogenesis up to 90 days post-injection at doses of 7.5 and 30 mg (59). In a Phase I dose escalation study with 12 months follow-up in ischemic cardiomyopathy, 17 subjects in New York Heart Association class III heart failure, with an ejection fraction ≤40% on stable medical therapy, were enrolled to receive 5, 15, or 30 mg of plasmid SDF-1 via endomyocardial injection. The primary end points for safety and efficacy were at 1 and 4 months, respectively. The primary safety end point was a major adverse cardiac event while efficacy end points were changes in quality of life, New York Heart Association (NYHA) class, 6-min walk distance, single photon emission computed tomography, N-terminal pro-brain natruretic peptide, and echocardiography at 4 and 12 months. The primary safety end point was met. At 4 months, all of the cohorts demonstrated improvements in 6-min walk distance, quality of life, and NYHA class (93). Stromal cell-derived factor-1 plasmid treatment for patients with heart failure (STOP-HF) was a Phase II, double-blind, randomized, placebo-controlled trial to evaluate safety and efficacy of a single treatment of plasmid SDF-1 delivered via endomyocardial injection to patients with ischemic heart failure. The primary endpoint was a composite of change in 6 min walking distance and Minnesota Living from Heart Failure Questionnaire from baseline to 4 months follow-up. The primary endpoint was not met (*P* = 0.89). For the patients treated with pSDF-1, there was a trend toward an improvement in left ventricular ejection fraction at 12 months (placebo vs. 15 vs. 30 mg ΔLVEF: −2 vs. −0.5 vs. 1.5%, *P* = 0.20). Patients in the first tertile of EF (<26%) that received 30 mg of pSDF-1 demonstrated a 7% increase in EF compared with a 4% decrease in placebo (ΔLVEF = 11%, *P* = 0.01) at 12 months (94). Although the reasons for the overall failure are currently unclear, the differential benefit in those with advanced left ventricle dysfunction raises the possibility of differential mechanisms that would be operational in more advanced patients. These include the possible overexpression of CXCR4 in cardiac myocytes in the infarct border leading to a negative inotropic state (95). The transient overexpression of SDF-1 in ischemic cardiomyopathy has been shown to lead to long-term down-regulation of cardiac myocyte CXCR4 expression, re-recruiting the contractile function of the border zone (61). Patients with greater left ventricle dysfunction are likely to have a greater volume of myocardial tissue under stress; therefore, a greater demonstrable response to SDF-1 overexpression. Another important reason could be that the upregulation of DPP4 in the border zone of the infarct or around ischemic areas may have resulted in rapid degradation of SDF-1 limiting the efficacy of such an approach. Since both the Phase I and Phase II studies were performed in the absence of DPP4 inhibition, it could be speculated that the results may have been different if the trials had been performed either in the presence of a DPP4 inhibitor.

## Conclusion and Future Directions

Due to the importance of SDF-1/CXCR4 axis in the stem cell and progenitor cell survival and function, understanding this axis and molecules that modulate their production and action will be of utility for the treatment of cardiovascular disease. There are a number of clinically approved drugs, including DPP4 inhibitors and parathyroid hormone, which have the ability to enhance SDF-1/CXCR4 responsiveness and may improve the outcome of cardiovascular diseases. Several recent large scale clinical trials have indicated that unlike most other oral anti-diabetic drugs that promote cardiovascular disease, DPP4 inhibitors are safe from cardiovascular standpoint despite lack of evidence showing beneficial effect (96–98). To what extent SDF-1/CXCR4 axis contributes to this effect requires further investigation.

## Conflict of Interest Statement

The authors declare that the research was conducted in the absence of any commercial or financial relationships that could be construed as a potential conflict of interest.

## Funding

This work was supported by grants from AHA (15SDG25700381 and 13POST17210033) and Mid-Atlantic Nutrition Obesity Research Center (NORC Pilot & Feasibility Program) to Dr. Zhong.
